# Microarray profiling of miRNA and mRNA expression in *Rag2* knockout and wild-type mouse spleens

**DOI:** 10.1038/sdata.2017.199

**Published:** 2018-01-09

**Authors:** Abu Musa Md Talimur Reza, Seong-Keun Cho, Yun-Jung Choi, Kwonho Hong, Jin-Hoi Kim

**Affiliations:** 1 Department of Stem Cell and Regenerative Biotechnology, Humanized Pig Research Center (SRC), Konkuk University, Seoul 143-701, Republic of Korea; 2 Department of Animal Science, Busan National University, Miryang, Gyeongnam 50463, Republic of Korea

**Keywords:** miRNAs, Transcriptomics, Disease model, Microarray analysis, Immunology

## Abstract

The **Rag2** knockout (KO) mouse is one of the most popular immune compromised animal models used in biomedical research. The immune compromised state concurrently alters many signalling pathways and molecules, including miRNAs and mRNA transcripts that are involved in important biological processes. In addition, miRNAs and transcripts are interdependent, often forming a feedback loop; dysregulation in one might alter the expression of the other, and both participate in many physiological processes including immune regulation. Here, we describe a comprehensive dataset containing alterations in the expression of both miRNAs and mRNAs in *Rag2* KO mice compared to their wild type counterparts. The miRNA and mRNA expression profiles were generated from total RNA using a miRNA expression microarray or a BeadChip microarray, respectively. Hence, this dataset will provide the groundwork for a comparative study of the miRNAs and mRNAs that are dysregulated in *Rag2* KO mice. It is hoped that the data will illuminate how miRNAs mediate immune regulation, as well as the interaction between miRNAs and mRNAs in *Rag2* KO mice.

## Background and Summary

Due to the leaky characteristics (especially at older ages) of many severe combined immune deficiency (SCID) animals^1^, alternative mice strains with more stable defective immune system have been developed, including the *Rag1* and *Rag2* knockout (KO) mice^2,3^. At the present, *Rag2* KO mice are a popular animal model because of their consistent immune compromised status. The *Rag2* gene encodes a protein that regulates the V(D)J recombination processes during the biogenesis of B- and T-cells. Functionally, the *Rag2* and Rag1 proteins form a complex, which is able to create double-strand breaks by cleaving DNA at conserved recombination signal sequences and thus, contribute to B- and T-cell development. Due to this essential role, depletion of the *Rag2* gene successfully impairs the development of both B- and T-cells^3^.

The deposited dataset offers an opportunity to investigate the global changes in miRNAs and mRNA expression in the *Rag2* KO mice. MicroRNAs (miRNAs) are non-coding small RNA molecules that post-transcriptionally regulate the expression of approximately one third of human genes either by inducing mRNA degradation or by inhibiting translation^4,5^. It is known that miRNAs are involved in a variety of biological and physiological processes, and that the expression of miRNAs and genes are influenced by each other, meaning that changes in one can regulate changes in the other. Evidence has shown that miRNAs could alter genes expression and signalling in the immune system, and regulate different biological and cellular processes^6–10^. Hence, it has become a necessity to understand the miRNA and mRNA expression profiles simultaneously using a holistic approach. Hence, the current dataset opens the groundwork to compare the interactions between miRNAs and mRNAs in an immune compromised state. It might also identify the miRNAs that are important in the immune regulatory processes.

**Figure 1:**
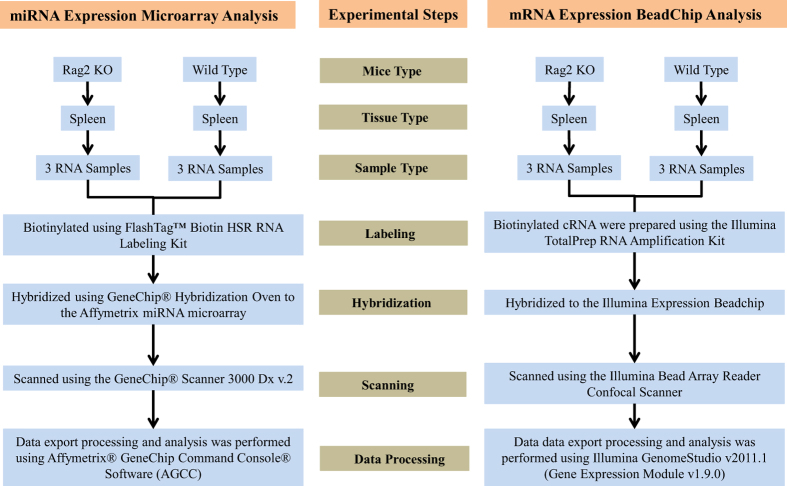
Flow chart illustrating the steps for miRNA expression microarray analysis and gene expression BeadChip analysis.

As shown in Fig. 1, in this study we obtained the spleen from three *Rag2* KO mice and three wild type mice, and then RNA samples were prepared from the spleen tissue for the analysis of their miRNAs profiles using Affymetrix Genechip miRNA 4.0 arrays. In parallel, mRNA expression profiling was performed using the Illumina MouseRef-8 v2 Expression BeadChip platform. The Affymetrix Genechip miRNA 4.0 array offers updated content without compromising the high performance of the previous-generation arrays, and it also provides a comprehensive coverage that is designed to interrogate all mature miRNA sequences in miRBase Release 20. In addition, the miRNA results can be easily analysed since the analysis files contain the host gene ID, predicted and validated miRNA target genes, and clustered miRNA information. In contrast, the Illumina MouseRef-8 v2.0 BeadChip Kit content was derived from the National Center for Biotechnology Information reference sequence (NCBI RefSeq) database (Build 36, Release 22). The chip was supplemented with probes derived from the mouse exonic evidence based oligonucleotide (MEEBO) set, as well as standard protein-coding sequences described in the RIKEN FANTOM2 database. In addition, the MouseRef-8 v2.0 BeadChip targets approximately 25,600 well-annotated RefSeq transcripts, over 19,100 unique genes, and enables the interrogation of eight samples in parallel. The MouseRef-8 v2.0 BeadChip Kit uses the DirectHyb assay and is compatible with the iScan, HiScan, and Bead array reader systems. Although, the recent mouse genome reference is GRCm38 having higher number of genes (around 45,300 genes), but the data could be reanalysed using the raw datasets that is deposited at Gene Expression Omnibus (GEO). In addition, the deposited datasets showed some inter-individual variation among mice in the expression of both miRNAs and mRNAs, especially the inter-individual variability of miRNAs expression is little higher. This is might be the results of either or both individual variation and technical variation. In this datasets, microarray analysis did not have technical replication, but there were technical replications during the qRT-PCR validation experiments. As well, the samples used for qRT-PCR experiment were from different individual than the individual used for microarray analysis. The validation of selected miRNAs and mRNAs expression overall reflected the microarray data. And the variation found among the mice could be the result of individual variation.

## Methods

### Animals

The experimental mice were maintained on a congenic C57Bl/6J background and were allowed access to standard mouse chow (Cargill Agri Purina, Inc., Seongnam-Si, Korea) and water on an *ad libitum* basis. Spleen tissues, collected from approximately one-year-old *Rag2* KO and wild type mice were used in this study. Upon excision from the mouse, the spleen tissue was placed in appropriately labelled 1.5-ml Eppendorf tube and stored at −80 °C until use. The animal experiments were conducted according to the guidelines of the Konkuk University Animal Care and Experimentation Community (IACUC approval number: KU12045). *Rag2* KO mice were originally purchased from Taconic Biosciences, Inc. (Hudson, NY, USA).

### RNA isolation

Total RNA (including miRNAs) was isolated from mouse spleen tissue using the miRNeasy mini kit (Qiagen, Valencia, CA, USA) following the manufacturer's protocol. Briefly, for each sample, around 30 mg of spleen tissue was placed in a 2-ml collection tube containing 700 μl of QIAzol Lysis Reagent. Following this, homogenization was performed immediately using the TissueLyser LT until the sample had become uniformly homogeneous (around one minute) and the traces of the tissue had disappeared. After this, the homogenate was incubated at RT (15–25 °C) for 5 min, and then, 140 μl chloroform was added to each tube containing the homogenate, capped, shaken vigorously for 15 s, and incubated at RT for another 2–3 min. The lysate was then centrifuged for 15 min at 12,000×*g* at 4 °C. After centrifugation, the upper aqueous phase was transferred to a new collection tube and supplemented with 1.5 volumes (around 525 μl) of 100% ethanol and mixed thoroughly by inverting the tube several times.

Up to 700 μl of the lysate (including any precipitate) was transferred to an RNeasy Mini spin column in a 2-ml collection tube and gently centrifuged at ≥8,000×*g* for 15 s at RT. The flow-through was discarded and 700 μl of buffer RWT was added to the RNeasy Mini spin column and centrifuged for 15 s at ≥8,000×*g* to wash the column. The flow-through was discarded and 500 μl buffer RPE was added to the RNeasy Mini spin column and centrifuged for 15 s at ≥8,000×*g* to wash the column. Again, the flow-through was discarded and another 500 μl buffer RPE was added to the RNeasy Mini spin column and centrifuged for 2 min at ≥8,000×*g*, and the collection tube containing the flow-through was discarded. The RNeasy Mini spin column was placed into a new 2-ml collection tube and centrifuged in a micro centrifuge at 12,000×*g* for one minute to dry the column membrane. Finally, the RNeasy Mini spin column was transferred to a new 1.5-ml collection tube and 30–50 μl RNase-free water was added directly onto the column membrane, incubated for one minute, and then centrifuged for 2 min at ≥8,000×*g* to elute the RNA. The isolated RNA was stored at −80 °C for downstream analysis.

### miRNA expression microarray analysis

#### 1. Affymetrix miRNA arrays methods

For miRNA expression analysis, the Affymetrix Genechip miRNA 4.0 array was used according to the manufacturer's instructions. For each sample, 1 μg of RNA was labelled using the FlashTag™ Biotin RNA Labeling Kit (Genisphere, Hatfield, PA, USA). Following this, the labelled RNA was quantified, fractionated, and hybridized to the miRNA microarray following a series of consecutive steps. First, the labelled RNA samples were heated to 99 °C for 5 min followed by heating at 45 °C for another 5 min. After this, hybridization with the RNA-array was performed with continuous agitation at 60 rpm for 16 h at 48 °C on an Affymetrix® 450 Fluidics Station. The miRNA microarray chips were then washed and stained using the Genechip Fluidics Station 450 (Affymetrix, Santa Clara, CA, USA). Finally, the miRNA microarray chips were scanned using an Affymetrix GCS 3,000 scanner (Affymetrix, Santa Clara, CA, USA) and the signal values were evaluated using the Affymetrix® GeneChip™ Command Console software.

#### 2. Raw data preparation and statistical analysis

Raw data were automatically extracted using the Affymetrix data extraction protocol in the Affymetrix GeneChip® Command Console® Software (AGCC). CEL file import, miRNA level RMA+DABG-All analysis, and export of the results were all performed using Affymetrix® Expression Console™ software. A comparative analysis between the test samples and the control samples was carried out using fold-change and an independent *t*-test, in which the null hypothesis was ‘there is no difference between the groups'. The false discovery rate (FDR) was controlled by adjusting the *p* value using the Benjamini-Hochberg algorithm. Hierarchical clustering was performed using complete linkage and Euclidean distance as a measure of similarity for the differentially expressed miRNAs. Statistical tests and visualization of differentially expressed miRNAs were conducted according to the R statistical language v. 3.1.2. (www.r-project.org).

### BeadChip microarray analysis for transcriptomic profiling

#### 1. Labelling and purification

Total RNA samples were amplified and purified using the Ambion Illumina RNA amplification kit (Ambion, Austin, USA) to yield biotinylated cRNA following the manufacturer’s protocol. For each sample, 550 ng of total RNA was reverse-transcribed to cDNA using a T7 oligo (dT) primer. Following this, second-strand cDNA was synthesized, *in vitro* transcribed, and labelled with biotin-NTP. After this, the purified cRNA was quantified using a ND-1,000 Spectrophotometer (NanoDrop, Wilmington, DE, USA).

#### 2. Hybridization and data export

For each sample, 750 ng of labelled cRNA sample were hybridized to each Mouse Ref-8 expression v.2 bead array for 16–18 h at 58 °C, as per the manufacturer’s protocol (Illumina, Inc., San Diego, CA, USA). Following this, the array signals were detected using Amersham fluorolink streptavidin-Cy3 (GE Healthcare Bio-Sciences, Little Chalfont, UK) according to the bead array manual. Finally, the arrays were scanned with an Illumina bead array Reader confocal scanner following the manufacturer's instructions.

#### 3. Raw data preparation and statistical analysis

The quality of hybridization and overall performance of the BeadChips were monitored by visual inspection of both the internal quality control checks and scanned raw data. The raw data were extracted using Illumina GenomeStudio v2011.1 (Gene Expression Module v1.9.0) software. The array probes were then transformed by logarithm and normalized by the quantile method. Statistical significance of the expression data was determined using both fold change and a local pooled error (LPE) test with the null hypothesis being ‘there are no differences between the groups’. The FDR was controlled by adjusting the *p* values using the Benjamini-Hochberg algorithm.

Hierarchical cluster analysis was performed using complete linkage and Euclidean distance as a measure of similarity for the differentially expressed genes. Gene-enrichment and functional annotation analysis for the significant probe list was performed using DAVID (http://david.abcc.ncifcrf.gov/home.jsp). All data analysis and visualization of differentially expressed genes was conducted using R 3.0.2 (www.r-project.org).

### Verification of the dysregulated expression of selected miRNAs by qRT-PCR

For verification of the chip data, the expression levels of miRNAs were detected using quantitative real-time reverse transcription PCR (qRT-PCR) according to the instructions provided with the Mir-X miRNA qRT-PCR SYBR kit (Clontech Laboratories, Inc., CA, USA). Briefly, a single-step polyadenylation and reverse-transcription reaction was carried out to prepare cDNAs using RNA samples derived from *Rag2* KO and wild type mice spleen (different individuals than microarray). For qRT-PCR analysis, specific sequences of miRNAs were regarded as miRNA-specific 5′ primers, and the mRQ 3′ primers provided with the kit were used as the 3′ primers for all miRNAs. The U6 RNA was used to normalize the threshold cycle (Ct) values, and miRNAs expression was quantified using the relative quantitation method (2^−ΔΔCt^).

### Verification of the dysregulated expression of selected genes by qRT-PCR

For the verification of dysregulated mRNA expression, cDNA was synthesized from the total RNA (extracted from different individuals than the microarray experiments) using the QuantiTect Reverse Transcription Kit (Cat No. 205313; Qiagen, USA) according to the manufacturer’s protocol. Briefly, genomic DNA was eliminated by a reaction in the first step using gDNA wipe out buffer provided with the QuantiTect Reverse Transcription Kit and then cDNA was synthesized during the second step. The expression of selected genes in the *Rag2* KO and wild type samples was detected by SensiFast SyBR Lo-ROX Kit (BIO-94003; Bioline, UK). GAPDH was used to normalize the threshold cycle (Ct) values, and gene expression was quantified using the relative quantitation method (2^−ΔΔCt^).

## Data Records

CEL and CHIP files associated with the samples analysed in this study are deposited at GEO with the accession number GSE102941 (Data Citation 1) for miRNA expression microarray and GSE103230 (Data Citation 2) for mRNA expression BeadChip array. The metadata record regarding the samples is provided in Table 1 and Table 2, respectively.

## Technical Validation

### Sample preparations and quality control

To minimize the technical errors several precautionary steps were taken into consideration. For example, the control mice used for this experiment were from same genetic background (C57Bl/6J), and the mice were selected randomly (both *Rag2* KO and wild type) for experiments. In addition, each group contained three animals to avoid any misinterpretation due to biological variation. To avoid any unintended alterations in miRNA or gene expression arising as a result of sacrificing the mice, the collected spleen tissues were stored directly in a −196 °C liquid nitrogen tank until used for downstream analysis. In addition, to avoid contamination, the dissecting tools were properly cleaned between tissue extractions from different mice. RNA was also isolated following the instructions provided with the isolation kit, and only high quality RNA was used for experiments. For RNA quality control, its purity and integrity were evaluated using the OD 260/280 ratio, and RNA integrity number (RIN) was analysed using an Agilent 2,100 Bioanalyzer (Agilent Technologies, Palo Alto, CA, USA).

### Quality check of microarray analysis

**Figure 2:**
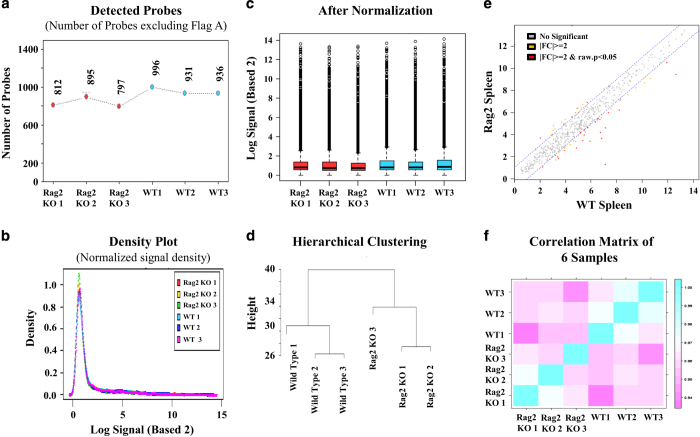
Quality check of miRNA microarray expression data. (**a**) Graph showing the number of miRNA probes detected in each sample. (**b**) Density plot showing the distribution of each sample. (**c**) Box plot showing the distribution of maximum, minimum, and percentile values for the normalized signal of each sample. (**d**) Hierarchical clustering analysis among the samples. (**e**) Scatter plot showing the expression level of miRNAs in *Rag2* KO versus wildtype spleen (**f**) Correlation matrix showing the degree of repeatability between samples.

**Figure 3:**
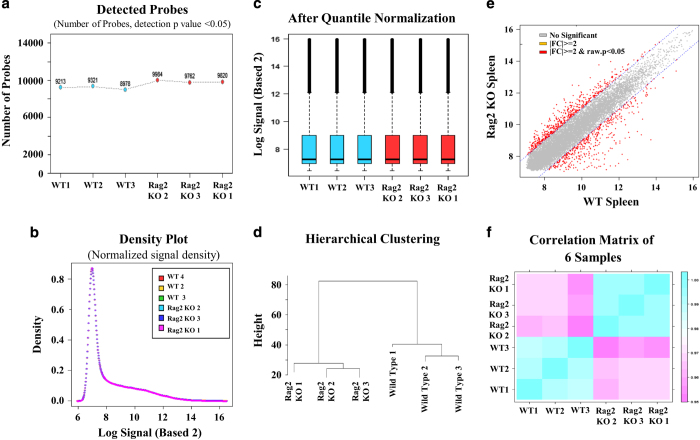
Quality check of BeadChip gene expression data. (**a**) Graph showing the number of gene probes detected in each sample. (**b**) Density plot showing the distribution of each sample. (**c**) Box plot showing the distribution of maximum, minimum, and percentile values for the normalized signal of each sample. (**d**) Hierarchical clustering analysis among the samples. (**e**) Scatter plot showing the expression level of mRNAs in *Rag2* KO versus wildtype spleen. (**f**) Correlation matrix showing the degree of repeatability between samples.

**Figure 4:**
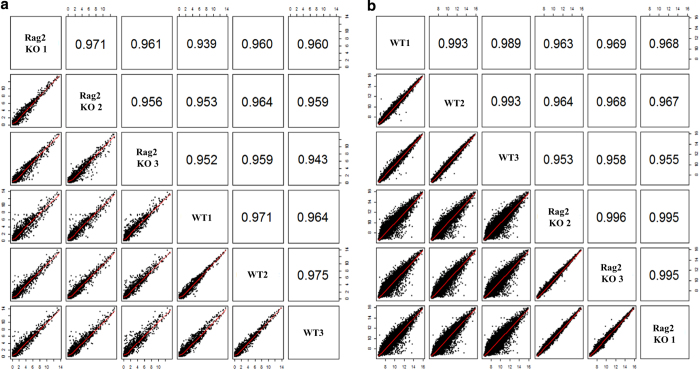
The principal component analysis (PCA) showing the repeatability among samples. (**a**) The scatter plot showing the repeatability of miRNAs expression among the samples of *Rag2* KO and wildtype mice. (**b**) The scatter plot showing the repeatability of mRNAs expression among the samples of *Rag2* KO and wildtype mice.

As shown in Fig. 2, the quality of the miRNA expression microarray analysis was checked by analysing the miRNA plots for each sample (Fig. 2a), by preparing density and QC matric line plots (Fig. 2b), by analysing the distribution of the maximum, the minimum, and percentile values for the normalized signal of each sample (Fig. 2c), and by performing the hierarchical clustering and measuring correlation matrix between samples (Fig. 2d–f). In contrast, the quality of the gene expression data obtained from the BeadChip array was ensured by detecting the mRNA plots for each sample (Fig. 3a) by drawing a density plot to measure the distribution of each sample (Fig. 3b), by measuring the distribution of the maximum, minimum, and percentile values for the normalized signals (Fig. 3c), and by performing the hierarchical clustering and measuring the correlation matrix between samples (Fig. 3d–f). As shown in Fig. 4, the degree of repeatability among the samples was further measured in both types (miRNA and mRNA) of microarray analysis using principal component analysis (PCA).

### Verification of microarray data by qRT-PCR

**Figure 5:**
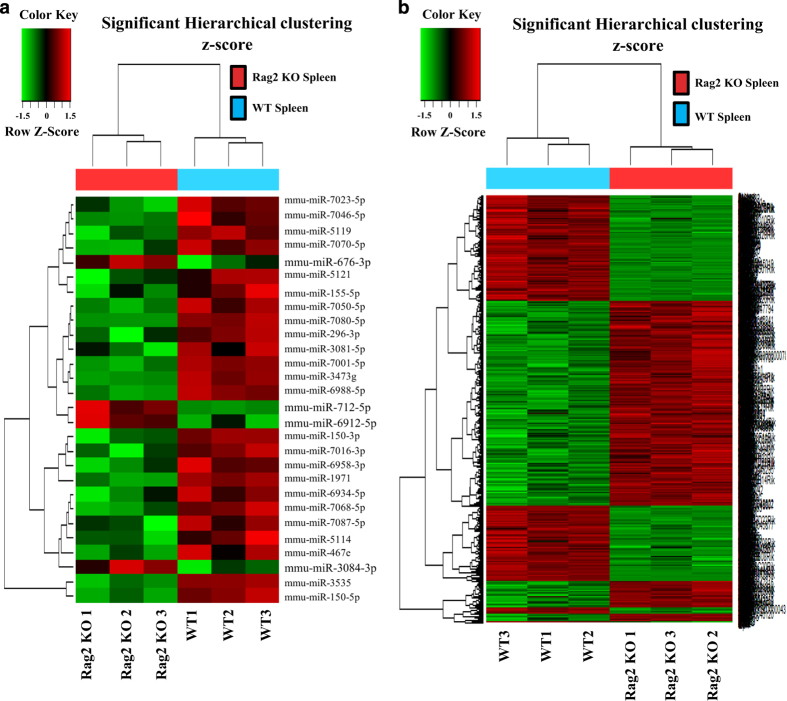
Heat map showing the differential expression of miRNAs and genes in *Rag2* KO and wild type mice spleens. (**a**) Hierarchical clustering showing the differential expression of miRNAs in *Rag2* KO and wild type mice spleens. (**b**) Hierarchical clustering showing the differential expression of genes in *Rag2* KO mice and wild type spleens.

**Figure 6:**
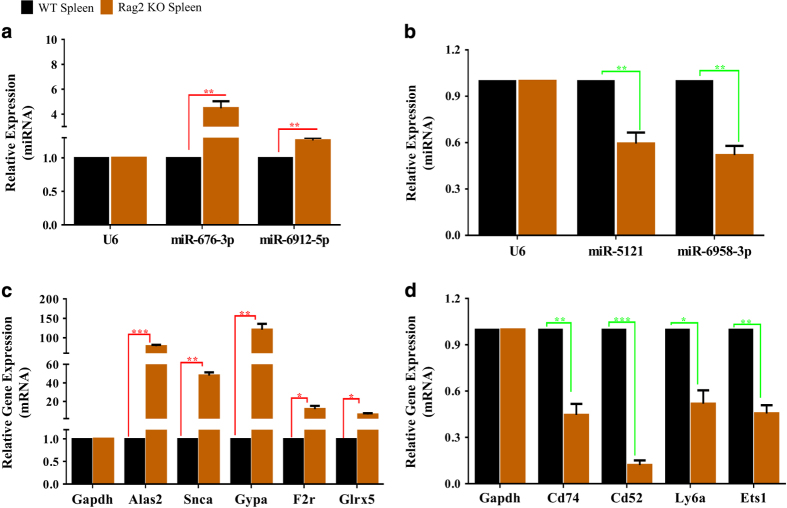
qRT-PCR validation of the dysregulated expression of selected miRNAs and genes in *Rag2* KO mice spleen. (**a**) Bar diagram showing the upregulated expression of selected miRNAs in *Rag2* KO mice spleens compared to wild type mice spleens. (**b**) Bar diagram showing the downregulated expression of selected miRNAs in *Rag2* KO mice spleens compared to wild type mice spleens. (**c**)Bar diagram showing the upregulated expression of selected genes in *Rag2* KO mice spleens compared to wild type mice spleens. (**d**)Bar diagram showing the downregulated expression of selected genes in *Rag2* KO mice spleens compared to wild type mice spleens. *p<0.05, **p<0.01, ***p<0.001.

As shown, twenty-eight miRNAs (Fig. 5a) and 936 genes (Fig. 5b) showed two-fold or more changes in expression between *Rag2* KO mice and their wild type counterparts. To verify the authenticity of these microarray analyses, qRT-PCR was performed to confirm the expression of these differentially expressed miRNAs and genes. For these experiments, a separate group of *Rag2* KO and wild type mice were used, which were maintained and raised under the same experimental conditions as the mice that were used for the miRNA expression microarray analysis and the BeadChip microarray analysis. The expression of several selected miRNAs (*mmu-miR-676-3p*, *mmu-miR-6912-5p*, *mmu-miR-5121* and *mmu-miR-6958-3p*), and genes (*Alas2*, *Snca*, *Gypa*, *F2r*, *Glrx5*, *Cd74*, *Cd52*, *Ly6a* and *Ets1*) were examined, and we were able to demonstrate that the expression of the selected miRNAs and mRNAs as shown by qRT-PCR agree with the microarray data obtained by either miRNA expression microarray or BeadChip array, respectively (Fig. 6).

## Usage Notes

The study of miRNAs and gene expression using microarrays has become one of the most widely used techniques in molecular biology^11^ and over the last decade has been used with great success for the detection of changes in molecular pathways and signalling networks^12,13^. Moreover, miRNAs are important signalling molecules, in themselves, that are involved in the regulation of the expression of numerous genes in different mammalian species^5^. Since *Rag2* KO mice are a popular immune-compromised model we believe that this dataset will provide the groundwork for understanding the dysregulation of the expression of genes and miRNAs in *Rag2* KO mice. The data could also provide important findings about the involvement of miRNAs during immune regulation, as well as identifying miRNAs that are directly or indirectly involved in immune regulation in mammals.

One major advantage of this study is that both the miRNA and gene expression profiles have been performed simultaneously. It is hoped that this will enable the user to investigate more precisely the interaction between miRNAs and genes in *Rag2* KO mice. It could also provide some answer to the question of how miRNAs are involved in immune regulation and gene expression in *Rag2* KO mice. In addition, the data could be used to confirm the accuracy of *in silico* predicted targets of particular dysregulated miRNAs.

Finally, many publicly available databases can now be used for the analysis of both miRNA expression microarray data and gene expression BeadChips array data. These databases include but are not limited to, DAVID, PANTHER, Gene Set Expression Analysis (GSEA), STRING, and Cytoscape. In addition, different analysis tools such as Bingo, ClueGO, CluePedia, cyTransFinder, GeneMania, MetDisease, MetScape, ReactomeFIPlugin as well as commercially available software such as QIAGEN's Ingenuity Pathway Analysis (IPA), could all be of use in analysing and interpreting the data. Overall, it is hoped that the deposited datasets will be useful in immunological and biomedical research.

## Additional information

**How to cite this article:** Reza, A. M. M. T. *et al.* Microarray profiling of miRNA and mRNA expression in *Rag2* knockout and wild-type mouse spleens. *Sci. Data* 5:170199 doi: 10.1038/sdata.2017.199 (2018).

**Publisher’s note:** Springer Nature remains neutral with regard to jurisdictional claims in published maps and institutional affiliations.

## Supplementary Material



## Figures and Tables

**Table 1:** Sample description for miRNAs expression microarray.

**Source**	**Background Strain**	**Protocol 1**	**Protocol 2**	**Protocol 3**	**Data**
WT Mouse1	C57Bl/6J	Spleen dissection	RNA extraction	miRNA expression microarray	GSM2750870
WT Mouse2	C57Bl/6J	Spleen dissection	RNA extraction	miRNA expression microarray	GSM2750871
WT Mouse3	C57Bl/6J	Spleen dissection	RNA extraction	miRNA expression microarray	GSM2750872
*Rag2* KO Mouse1	C57Bl/6J	Spleen dissection	RNA extraction	miRNA expression microarray	GSM2750867
*Rag2* KO Mouse2	C57Bl/6J	Spleen dissection	RNA extraction	miRNA expression microarray	GSM2750868
*Rag2* KO Mouse3	C57Bl/6J	Spleen dissection	RNA extraction	miRNA expression microarray	GSM2750869

**Table 2:** Sample description for mRNAs expression BeadChip array.

**Source**	**Background Strain**	**Protocol 1**	**Protocol 2**	**Protocol 3**	**Data**
WT Mouse1	C57Bl/6J	Spleen dissection	RNA extraction	mRNA BeadChip array	GSM2758547
WT Mouse2	C57Bl/6J	Spleen dissection	RNA extraction	mRNA BeadChip array	GSM2758548
WT Mouse3	C57Bl/6J	Spleen dissection	RNA extraction	mRNA BeadChip array	GSM2758549
*Rag2* KO Mouse1	C57Bl/6J	Spleen dissection	RNA extraction	mRNA BeadChip array	GSM2758544
*Rag2* KO Mouse2	C57Bl/6J	Spleen dissection	RNA extraction	mRNA BeadChip array	GSM2758545
*Rag2* KO Mouse3	C57Bl/6J	Spleen dissection	RNA extraction	mRNA BeadChip array	GSM2758546
