# Corrigendum: Microarray profiling of miRNA and mRNA expression in *Rag2* knockout and wild-type mouse spleens

**DOI:** 10.1038/sdata.2018.152

**Published:** 2018-07-31

**Authors:** Abu Musa Md Talimur Reza, Seong-Keun Cho, Yun-Jung Choi, Kwonho Hong, Jin-Hoi Kim

*Scientific Data* 5:170199 doi: 10.1038/sdata.2017.199 (2018); Published 9 January 2018; Updated 31 July 2018

The Data Descriptor incorrectly states that hybridization for the miRNA expression microarray analysis was performed with an Affymetrix 450 Fluidics Station. This step was performed using an Affymetrix GeneChip Hybridization Oven 645.

